# Carbon Nanodots Inhibit Tumor Necrosis Factor-α-Induced Endothelial Inflammation through Scavenging Hydrogen Peroxide and Upregulating Antioxidant Gene Expression in EA.hy926 Endothelial Cells

**DOI:** 10.3390/antiox13020224

**Published:** 2024-02-10

**Authors:** Jessica Chavez, Ajmal Khan, Kenna R. Watson, Safeera Khan, Yaru Si, Alexandra Y. Deng, Grant Koher, Mmesoma S. Anike, Xianwen Yi, Zhenquan Jia

**Affiliations:** 1 Department of Biology, University of North Carolina at Greensboro, Greensboro, NC 27412, USAa_khan10@uncg.edu (A.K.); y_si@uncg.edu (Y.S.); gfkoher@uncg.edu (G.K.);; 2 Chapel Hill High School, Chapel Hill, NC 27516, USA; fintryayd@gmail.com; 3 Department of Surgery, University of North Carolina at Chapel Hill, Chapel Hill, NC 27599, USA

**Keywords:** carbon nanodots, tumor necrosis factor-alpha, vascular inflammation, ROS

## Abstract

Carbon nanodots (CNDs) are a new type of nanomaterial with a size of less than 10 nanometers and excellent biocompatibility, widely used in fields such as biological imaging, transmission, diagnosis, and drug delivery. However, its potential and mechanism to mediate endothelial inflammation have yet to be explored. Here, we report that the uptake of CNDs by EA.hy926 endothelial cells is both time and dose dependent. The concentration of CNDs used in this experiment was found to not affect cell viability. TNF-α is a known biomarker of vascular inflammation. Cells treated with CNDs for 24 h significantly inhibited TNF-α (0.5 ng/mL)-induced expression of intracellular adhesion molecule 1 (*ICAM-1*) and interleukin 8 (*IL-8*). *ICAM-1* and *IL-8* are two key molecules responsible for the activation and the firm adhesion of monocytes to activated endothelial cells for the initiation of atherosclerosis. ROS, such as hydrogen peroxide, play an important role in TNF-α-induced inflammation. Interestingly, we found that CNDs effectively scavenged H_2_O_2_ in a dose-dependent manner. CNDs treatment also increased the activity of the antioxidant enzyme NQO1 in EA.hy926 endothelial cells indicating the antioxidant properties of CNDs. These results suggest that the anti-inflammatory effects of CNDs may be due to the direct H_2_O_2_ scavenging properties of CNDs and the indirect upregulation of antioxidant enzyme NQO1 activity in endothelial cells. In conclusion, CND can inhibit TNF-α-induced endothelial inflammation, possibly due to its direct scavenging of H_2_O_2_ and the indirect upregulation of antioxidant enzyme NQO1 activity in endothelial cells.

## 1. Introduction

Cardiovascular diseases (CVDs), commonly known as heart diseases, are diseases of the heart and blood vessels [1,2,3,4,5,6]. CVDs are the leading cause of death (18.6 million deaths annually) worldwide, hence making them a significant health and economic burden [1,7]. The United States alone spent USD 239.9 billion on CVDs in 2018–2019 [7]. According to the 2014 US mortality data, heart disease had the highest death rate for both sexes as well as across ethnicities. Currently, CVD remains the leading cause of death in the United States, with 695,000 deaths in 2021 [7,8,9].

Among the many forms of CVDs, atherosclerosis, which is characterized by the thickening of the blood vessels caused by plaque buildup in the inner lining of the blood vessels, is one primary form of CVDs [10,11,12,13,14]. Although the pathogenesis of atherosclerosis is complex, accumulating data show that inflammation and the subsequent dysfunction of endothelial cells play a fundamental role in the initiation and progression of atherosclerosis [11,12]. Pro-inflammatory molecules, including thrombin, tumor necrosis factor-alpha (*TNF-α*), macrophage chemoattractant protein-1 (*MCP-1*, also known as *CCL2*), interleukin 8 (*IL-8*), E-selectin, P-selectin, vascular cell adhesion molecule-1 (*VCAM-1*), and intracellular adhesion molecule-1 (*ICAM-1*) have been implicated in the development of atherosclerosis [15,16,17,18]. Studies show that TNFα is an important pro-inflammatory mediator. Endothelial inflammation is essential in developing atherosclerosis, and searching for agents that can inhibit the expression of inflammatory molecules in vascular endothelial cells is an effective way to prevent or treat CVD.

Recently, nanotechnology has received widespread attention in biomedicine. Carbon nanodots (CNDs) are spherical or quasi-spherical structured photoluminescent nanoparticles smaller than 10 nm. Due to its good water solubility and biocompatibility, these nanodots have broad application prospects in bioimaging, biosensing, and improved drug delivery [19,20,21,22,23,24]. CNDs have abundant surface functional groups, high solubility, and photoluminescence (PL), with emission ranging from UV to near-infrared light. CNDs PL is affected by the nanoparticle size and surface functional groups [25,26]. Different quantum yields (QY) of CNDs can be achieved by adjusting the synthesis route. Zhu et al. synthesized CNDs using citric acid and ethylenediamine. Surface passivation with molecules such as polyethylene glycol can improve QY, and its QY is as high as 80.6% [27,28,29]. Green synthesis using carbon precursors can produce CNDs with low toxicity and high quantum yield, making them promising candidates for biomedical applications such as therapeutic delivery and bioimaging. Efforts in CND synthesis have focused on fine tuning for potential nuclear translocation, an important aspect of cancer therapy, overcoming the challenge of CNDs remaining primarily in the cytoplasm [21,22]. Studies have also previously shown that CNDs can track biological processes inside cells [30], deliver doxorubicin to cancer target cells [31,32], and scavenge radicals during oxidative stress [33,34]. Zhang et al. used a microwave route to synthesize CNDs with citric acid and urea as precursors [35]. These same CNDs were analyzed for antioxidant activity through the nitrogen-centered 2-2-diphenyl-1-picrylhydrazyl radical (DPPH•) assay, which is a well-established assay used to measure antioxidant activity [36,37,38] by measuring the absorbance of DPPH•. The results showed that with increasing concentrations of CNDs, antioxidant activity increased, suggesting the potential bio-application of CNDs in scavenging free radicals [35].

Previous studies demonstrate that endothelial inflammation induced by TNF-α plays a fundamental role in the initiation and progression of atherosclerosis. The transcriptional regulation of cytokines and vascular adhesion molecules are responsible for leukocyte adhesion to activated endothelial cells [39,40]. However, the effect of CNDs on endothelium has not been reported; in particular, its potential role in TNF-α-induced endothelial inflammation needs to be explored. Human EA.hy926 endothelial cells are widely recognized for their expression of intercellular adhesion molecule 1 (*ICAM-1*), E-selectin, and other important endothelial cell surface biomarkers [41,42,43]. EA.hy926 is one of the best-characterized vascular endothelial cell lines. It has been used as a model for various vascular studies and screening of potential anti-atherosclerotic natural compounds such as Isoquercitrin and Shixiao San [41,42]. The main purpose of this study was to investigate the uptake and role of CNDs in the modulation of TNFα-induced inflammation in EA.hy926 endothelial cells. The ability of CNDs to scavenge hydrogen peroxide was also examined. This study provides new insights into the anti-inflammatory potential of CNDs and their potential therapeutic effect on inflammatory diseases, especially atherosclerosis.

## 2. Materials and Methods

### 2.1. Materials

EA.hy926 endothelial cells (CRL-2922 ™) from ATCC were purchased. For cell culturing, Dulbecco’s Modifies Eagles Medium (DMEM) was acquired from Gibco (12100-061 (Waltham, MA, USA)), and both the fetal bovine serum (FBS 26140-079) and penicillin–streptomycin (15140-122) were purchased from Gibco. For cell viability, thiazolyl blue tetrazolium bromide was bought from Sigma (298-93-1 (St. Louis, MO, USA)), and an Invitrogen (Waltham, MA, USA) molecular probe kit for flow cytometry was acquired (BMS306F1-300). PermaFluor Mountant for Imaging came from ThermoFisher Scientific (TA-006-FM (Waltham, MA, USA). For gene expression, the following reagents were purchased from Invitrogen: 10 mM dNTP (18427-013), random primers (S8875), M-MLV reverse transcriptase (28025-013), and 5X first strand buffer (Y02321). SYBR-Green was purchased from Applied Biosystems (4367659 (Waltham, MA, USA)), and primer sequences (see sequences in the qRT-PCR section) were purchased from Eurofins (Luxembourg). Apolipoprotein mice were obtained from Jackson Laboratory (002052 (Bar Harbor, ME, USA)).

### 2.2. Cell Culture

Human endothelial cells EA.hy926 were purchased from ATCC (Manassas, VA, USA) and grown in complete Dulbecco’s Modifies Eagles Medium (DMEM) containing 10% fetal bovine serum and 1% penicillin–streptomycin. Cells were cultured in 72 cm^2^ Cellstar cell culture flasks and kept in a humidified incubator at 37 °C and 5% CO_2_. Cells were replenished with fresh media every two days; cells were passaged according to ATCC’s sub-culturing procedure when cells were 90% confluent.

### 2.3. Carbon Nanodot (CND) Synthesis and Characterization

Carbon nanodots (CNDs) were synthesized and characterized following our published method [44,45]. Briefly, CNDs were synthesized by mixing 0.96 g of citric acid (CA) and 1 mL of Ethylenediamine (EDA) in 1 mL of deionized water. The solution was heated in a microwave synthesizer (CEM Corp 908005 (Matthews, NC, USA)) at 300 W for 10 min. To purify the CNDs, the solid was dissolved in DI water and dialyzed through a dialysis membrane with MWCO (molecular weight cut out) of 1000 Da for 24 h. The structure of CNDs was characterized using Fourier transform infrared spectroscopy (FTIR, Varian 670 (Palo Alto, CA, USA), and the size and morphology were characterized by atomic force microscopy (AFM, Agilent 5600LS (Santa Clara, CA, USA)). X-ray diffraction (XRD, Agilent Technologies Oxford Gemini, The Woodlands, TX, USA) was used to analyze the crystal structure of the CNDs. To study the chemical content and the structure of the CNDs, Fourier transform infrared spectroscopy (FTIR) was performed. The fluorescence intensity peaks were measured using a Cary fluorescence spectrophotometer to find the emission and excitation wavelengths for the CNDs. The CND concentration in distilled water for analysis was 0.06 mg/mL. The surface chemistry of CNDs was studied using carbon 1s X-ray photoelectron spectroscopy (XPS, ESCALAB 250 Xi, Thermo Fisher, West Sussex, UK).

### 2.4. CND Treatments

EA.hy296 cells were treated with CNDs with various concentrations for 24 h in Hank’s Balance Salt Solution (HBSS) media (pH, 7.4, containing glucose 1.0 g/L and. 0.35 g/L sodium bicarbonate without Ca^2+^, Mg^2+^). Before treating cells with CNDs, cells were rinsed with 7 mLs of 1X Phosphate-buffered Saline (PBS), the solution was decanted, and the treatment media was added.

### 2.5. CNDs Uptake Assay

Using the excitation and emission spectra of CNDs (the excitation and emission wavelengths of CNDs are 350 nm and 461 nm, respectively), a CND standard curve was created by measuring fluorescence at known concentrations of CNDs (0.005, 0.001, 0.05, 0.1, and 0.15 mg/mL) using a fluorescent plate reader (300 μL per standard). The associated data points are then plotted (CND concentration on the x-axis and fluorescence intensity on the y-axis), and a standard curve is generated. Cells were treated in a time-dependent and dose-dependent manner. When treatment was over, treatment media was decanted, and cells were rinsed twice with 7 mLs of 1X PBS. Cells were harvested using a cell scraper and centrifuged at 5000× *g* rpm for 5 min at 4 °C. The supernatant was decanted, and 900 µL of 1X PBS was used to suspend the cells. A black opaque 96-well plate was used to transfer 300 µL of suspended cells in technique triplicates. Fluorescence was then read using a Bio-Tek^®^ Synergy 2™ plate reader at 360/20 excitation and 460/20 emission. According to the standard curve, the concentration of CNDs (in the 300 μL PBS solution) can be calculated and then multiplied by the total 300 μL to obtain the total amount of CNDs for endothelial uptake.

### 2.6. Flow Cytometry Assay

Cells were treated in HBSS media and various concentrations of CNDs for 24 h; treatment media was collected, cells were trypsinized, and cold DMEM was used to neutralize trypsin and collected. Cells were centrifuged at 1000× *g* rpm at 4 °C for 5 min. Cells were washed with PBS, counted, and centrifuged in Eppendorf centrifuge tubes at 5000× *g* rpm at 4 °C for 5 min. The supernatant was decanted, and the resulting pellet was re-suspended in the appropriate volume of 1X binding buffer to produce a target cell concentration range of 1 × 10^5^ and 1 × 10^6^ cells per mL. In a flow cytometry tube, 100 µL of suspended cells were transferred, 5 µL of Annexin-V-FITC was added, followed by 1 µL of 7-AADD. Cells were kept on ice and incubated in the dark for 15 min. After incubation, 400 µL of the 1X binding buffer was added to the cells, and samples were analyzed using Guava^®^ EasyCyte Flow Cytometer (New York, NY, USA).

### 2.7. qRT-PCR

EA.hy926 cells were treated with CND only and 0.5 ng/mL TNF-α co-treatments. Cells were rinsed after treatment, and 1 mL of RNA isolation reagent was added to the treatment plate. Cells and solution were then transferred to a microcentrifuge tube and incubated for 5 min at room temperature (RT). After incubation, 200 µL of chloroform was added, followed by a 3 min RT incubation. The RNA extraction supernatant was spun at 12,000 rcf for 15 min at 4 °C. The aqueous phase was transferred to another tube, and 500 µL of isopropanol was added and incubated for 10 min RT. The supernatant was centrifuged at 12,000× *g* rcf for 10 min at 4 °C. The supernatant was decanted, and the RNA pellet was washed twice with 1 mL 75% ethanol and spun at 7400 rcf for 5 min at 4 °C. On the last wash, the supernatant was decanted, and the tube was inverted to air dry for 10 min. The RNA pellet was eluted in 15 µL of DEPC water and incubated for 20 min RT. RNA concentration was quantified, and cDNA was made after RNA was diluted to 500 ng/µL. Gene primer master mix was made, cDNA was diluted to a 1:9 ratio with DEPC water, 19 µL of the gene master mix was used per well, and 1 µL of cDNA was used to plate.


**Primers and Sequences**

**Target Gene**

**Forward**

**Reverse**

*GAPDH*
5′-CGACCACTTTGTCAAGCTCA-3′5′-AGGGGTCTACATGGCAACTG-3′
*ICAM-1*
5′-GGCTGGAGCTGTTTGAGAAC-3′5′-ACTGTGGGGTTCAACCTCTG-3′
*IL-8*
5′-TAGCAAAATTGAGGCCAAGG-3′5′-AAACCAAGGCACAGTGGAAC-3′

### 2.8. Cell Fixation CNDs

Cells were grown on coverslips and treated for 24 h with various CND concentrations in HBSS media. Coverslips were washed three times in 1X PBS twice and incubated in 3.7% PFA for 7 min. Coverslips were rinsed twice with 1X PBS following PFA fixation. To mount coverslips to slides, 15 µL of Epredia PermaFluor Mountant media (TA-006-FM (Waltham, MA, USA) was used. Slides were left overnight to set. Imaging was carried out using the Keyence BZ-X710 (Osaka, Japan) fluorescence microscope.

### 2.9. Hydrogen Peroxide Assay

Hydrogen peroxide (H_2_O_2_) was measured using a luminol-derived chemiluminescence (CL) assay as previously described [46]. Briefly, each microcentrifuge sample tube was added 10 μM luminol, 10 μM horseradish peroxidase, and 500 μM H_2_O_2_ in the presence or absence of 0.03 mg/mL, 0.3 mg/mL, and 0.6 mg/mL of CNDs in 1 mL of PBS mixture and maintained at 37 °C for 30 min. To check whether the assay is H_2_O_2_ specific, 500 U/mL catalase (CAT) was added in the presence of 10 μM luminol, 10 μM horseradish peroxidase, and 500 μM H_2_O_2_. Chemiluminescence was recorded as previously reported [46].

### 2.10. Antioxidant Sample Preparation

Treated EA.hy926 endothelial cells were harvested by decanting the media and rinsing twice with sterile 1X PBS. Cells were then trypsinized using 1.5 mL of trypsin and incubated for 3–5 min. Trypsin was inactivated by adding 7 mL of complete DMEM containing 10% fetal bovine serum; the collected cells were centrifuged at 1000× *g* rpm for 7 min at 4 °C. The pellet was suspended in 1 mL of PBS, transferred to a microcentrifuge tube, and spun at 5000 rpm for 5 min at 4 °C. PBS was decanted, and the pellet was suspended in 250 µL of tissue buffer (60 Mm K_2_HPO_4_/KH_2_PO_4_ + 1 mM EDTA pH 7.4 + 0.1% Triton-100). Cells were sonicated three times for 15 s with a 5 s resting period in between. The sonicated cells were spun at 13,000 rpm for 10 min at 4 °C. The antioxidant supernatant was transferred to a sterile microcentrifuge tube and stored at −80 °C.

### 2.11. Total Protein Assay

Pierce Coomassie (Bradford) Protein Dye was used for this assay, 800 µL of the dye was added to a test tube, followed by 6 µL of the antioxidant sample. For the standard, 10 µL of BSA 1.48 mg/mL was used, and the final volume in each test tube was 800 µL. Absorbance was read at 595 nm.

### 2.12. Total NADPH Quinone Oxidoreductase-1 (NQO1) Assay

Antioxidant sample lysate was used for the assay. A sensitive NQO1 reaction mixture was made using 12 mLs NQO1 Buffer (50 mM Tris-HCL, 0.08% TritonX-100 pH 7.5); 48 µL of 20 mM dichlorophenolindophenol (DCPIP) and 36 µL of 50 mM nicotinamide adenine dinucleotide phosphate (NADPH). In a cuvette, 698 µL of the NQO1 reaction mixture and 2 μL of antioxidant lysate sample were added, inverted, and immediately measured. Total NQO1 was obtained by reading the absorbance (600 nm every 15 s for 3 min) using a Beckman Coulter DU800 spectrophotometer (South San Franciso, CA, USA).

### 2.13. Glutathione Reductase (GR) Assay

Antioxidant lysate samples were used for this assay. In a cuvette, 465 µL of GR buffer (50 mM K_2_HPO_4_/KH_2_PO_4_ 1 mM EDTA pH 7.0), 60 µL of 20 mM GSSG, and 15 µL of the antioxidant sample (tissue buffer for blank) were added. The assay mixture was incubated for 3 min at 37 °C in the DU800 spectrophotometer. After incubation, 60 µL of 1.5 Mm NADPH was added, and cuvettes were inverted with parafilm and read at 37 °C, 340 nm, 30 s intervals for 5 min.

### 2.14. Glutathione S-Transferase

The light-sensitive GST reaction mixture was made with 10 mL GST buffer (0.1 M K_2_HPO_4_/KH_2_PO_4_ pH 6.5), 30 mg of bovine serum albumin (BSA), 200 µL of 50 mM 1-chloro-2,4-dinitrobenzene (CDNB) in ethanol, and 100 µL of 100 mM GSH. The DU800 spectrophotometer was blanked with a cuvette containing 585 µL of the reaction mixture and 15 µL of tissue buffer. Antioxidant lysate samples were used for this assay. Each cuvette for each sample contained 585 µL of the reaction mixture and 15 µL of the sample. The cuvette was inverted to mix the sample and read at 340 nm every 30 s for 5 min at 25 °C.

## 3. Results

### 3.1. Characterization of CNDs

**Figure 1:**
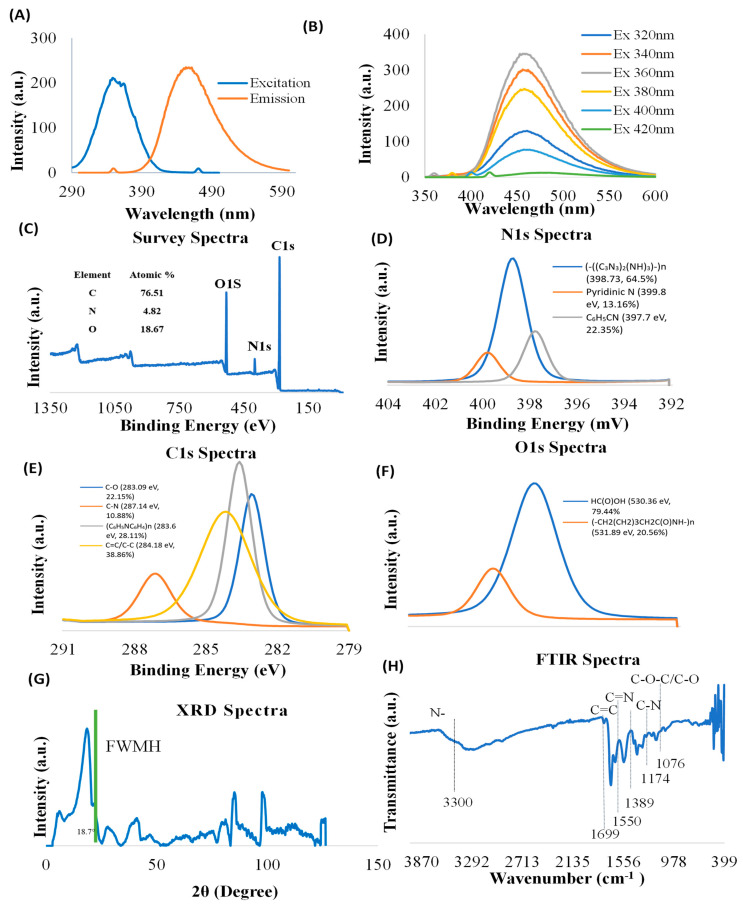
Characterization of CNDs. (**A**,**B**) Photoluminescence of CNDs was measured by Cary Eclipse TM Fluorescence Spectrophotometer. (**A**) The excitation intensity peak is at 350 nm, and the emission intensity peak is at 461 nm. (**B**) CNDs are excitation independent; the emission was analyzed after CNDs were tested at several excitation wavelengths. The highest emission is reached at 360 nm. (**C**–**F**) XPS Survey spectra of CNDs. (**G**) XRD Spectra of CNDs, and (**H**) FTIR Spectrum of CNDs.

Carbon nanoparticle synthesis provides unique features to nanomaterials. Characterization of CNDs, including photoluminescence properties, surface functional groups, and structure, is important for understanding their functional roles in biology. The nanoparticles used in this study possess their own fluorescence. Prior to conducting experiments, we verified the photoluminescence characterization of CNDs through ultraviolet–visible spectrophotometry (Figure 1A,B). The excitation and emission of CNDs were found to be 350 nm and 461 nm, respectively. To further verify the photoluminescence properties, we measured the emission CNDs for the following wavelengths: 320 nm, 340 nm, 360 nm, 380 nm, 400 nm, and 420 nm. Figure 1B shows that the emission peaks for different excitations are found at about 460 nm, suggesting that the synthesized CNDs are excitation independent.

The surface functional groups of CNDs were examined using XPS. The XPS survey spectra show characteristic peaks corresponding to C1s (283.89 eV), O1s (530.88 eV), and N1s (398.85 eV) (Figure 1C), confirming the presence of C, O, and N elements. The high-resolution N1s XPS spectrum exhibits three peaks located at 398.73 eV [(-((C_3_N_3_)_2_(NH)_3_)-)n, 64.5%], 399.8 eV [Pyridinic N, 13.16%], and 397.7 eV [C_6_H_5_CN, 22.35%] (Figure 1D). High-resolution C1s XPS spectra demonstrate that four components were detected at 283.09 eV [C-O, 22.15%], 287.14 eV [C-N, 10.88%], 283.6 eV [(C_6_H_5_NC_6_H_4_)n, 28.11%], and 284.18 eV [C=C/C-C, 38.86%] (Figure 1E). The results of deconvolution treatment for the high-resolution O1 s spectrum of the sample reveal two peaks centered at 530.36 eV [HC (O)OH, 79.44%] and 531.89 eV [(-CH_2_(CH_2_)_3_CH_2_C(O) NH-)n, 20.56%] (Figure 1F).

XRD was used to examine the crystallographic structure of CNDs. The XRD graph of CNDs reveals distinct peaks at various 2θ angles, indicating the crystalline structure of the material (Figure 1G). The highest peak was recorded at 2θ = 18.7°, which suggests a major crystallographic plane, with a Full Width at Half Maximum (FWHM) indicating the broadness of the peak. The comparatively narrower peak suggests higher crystallinity in our results, unlike amorphous forms of carbon dots [47].

Additional peaks were recorded at 2θ = 40°, between 80° and 90°, and near 100° signify the presence of other crystalline phases or orientations, contributing to the overall structural characteristics of the CNDs. However, the presence of these additional peaks shows that the CNDs were polycrystalline with other crystalline planes with distinct crystallographic features [48].

FTIR was used to characterize the structure of the CNDs. FTIR spectrum (Figure 1H) displays broad bonds at 3000–3600 cm^−1^, which are assigned to amine (N-H) and hydroxyl groups (OH). The N-H functional groups confer good hydrophilicity and stability of the CNDs in aqueous media [49]. The OH functional groups impart several properties to the molecule, including its polarity, water solubility, and ability to form hydrogen bonds with other molecules [50]. The bands at 1699 cm^−1^ originate from the vibrations of amide carboxyl (C=O) stretching. The bands at 1550 cm^−1^ are origin from the vibration of imine (C=N) stretching. The peak at 1174 cm^−1^ is ascribed to C-N bonds stretching in primary amines. Furthermore, the peak at 1076 cm^−1^ is attributable to either the C-O stretching vibrations or stretching C-O-C vibrations.

### 3.2. CNDs Uptake in EA.hy926 Endothelial Cells

**Figure 2:**
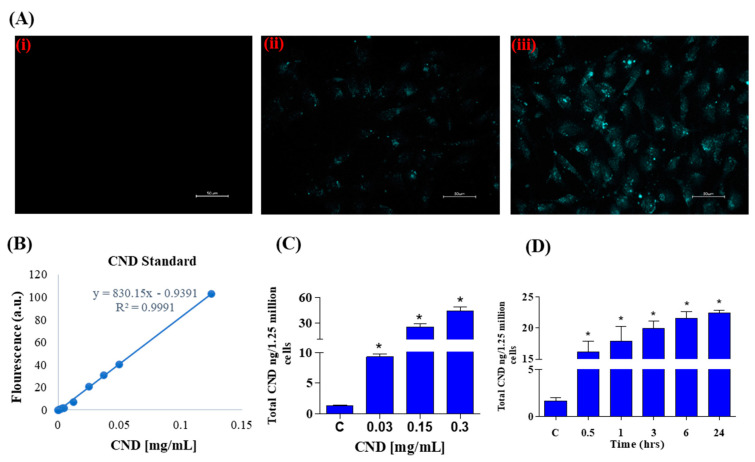
(**A**) Fluorescence in EA.hy926 human endothelial cells treated with increasing concentrations of CNDs after 24 h exposure. Panel (**i**–**iii**) shows an increase in fluorescence with increasing concentrations: (**i**): control; (**ii**): 0.3 mg/mL; (**iii**): 0.6 mg/mL. Cells were imaged using a Keyence microscope using a scale of 50 µm. (**B**) CNDs standard curve used to quantify CNDs uptake. The fluorescence of known concentrations of CNDs was measured in a microplate reader, and a line of regression was created to calculate nanomaterial uptake in cells. (**C**) EA.hy926 endothelial cellular uptake of CNDs. EA.hy926 endothelial cells were treated with CNDs in HBSS media for 24 h. (**D**) Time-dependent cellular uptake of CNDs. Time-dependent analysis of CND uptake of endothelial cells exposed to 0.15 mg/mL fluorescence was quantified via Bio-Tek Synergy 2.0 microplate reader. Nanodot uptake was calculated through the use of a standard curve. All data represent mean ± SEM. * *p* < 0.05 compared to the control group (n = 3).

Photoluminescent characterization was important for our experiments to study CND uptake using fluorescence microscopy. Using the excitation and emission spectra of CNDs, fluorescence imaging was carried out after a 24 h exposure of 0, 0.3, and 0.6 mg/mL CNDs in EA.hy926 endothelial cells. The DAPI channel was used to observe fluorescence in EA.hy926 cells after CND exposure. As expected, the control group (Figure 2A, panel i) does not have fluorescence under the DAPI channel. The cells treated with 0.3 and 0.6 mg/mL CNDs for 24 h showed fluorescence, indicating that the nanomaterial goes into the cells (Figure 2A, panels ii and iii). Before quantifying the number of CNDs that enter the cells, a carbon nanodot standard curve was created by measuring the fluorescence of known concentrations (Figure 2B). A regression line was created and used to calculate CND uptake. As shown in Figure 2C, EA.hy926 cells were treated with various concentrations of CNDs for 24 h, and uptake was quantified. CND uptake with the tested concentrations was found to be dose dependent. CND uptake analysis shows that nanomaterial uptake is also time dependent (Figure 2D).

### 3.3. Cell Viability Determined by Flow Cytometry

**Figure 3:**
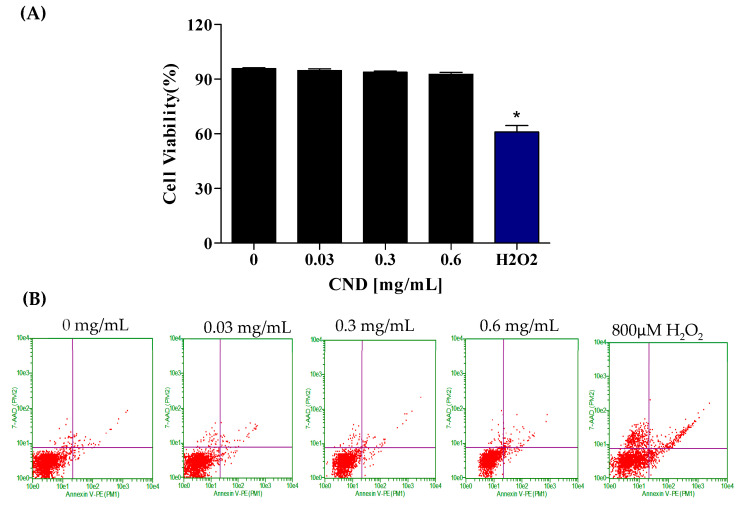
Cell viability was analyzed through the Annexin-FITC flow cytometry assay after 24 h of exposure. EA.hy926 cells were treated with HBSS media for 24 h for the (left to right) control, 0.03 mg/mL, 0.3 mg/mL, 0.6 mg/mL, and 800 µM H_2_O_2_ as the positive control treatments. (**A**) Flow cytometry plots. (**B**) Quantified cell viability graphs. Data shown are mean ± SEM (n = 3). * *p* < 0.05 compared to the control group (n = 3).

To further confirm the effect of CNDs on cell viability, the Annexin-FITC flow cytometry assay was used to analyze cells undergoing apoptosis and necrosis. Cells undergoing apoptosis are identified through the binding of the Annexin V-FITC conjugate to phosphatidylserine, a phospholipid cell membrane component normally located in the inner leaflet of the membrane that translocates to the outer surface during apoptosis. The CND concentrations used to analyze cell viability were 0 mg/mL, 0.03 mg/mL, 0.3 mg/mL, and 0.6 mg/mL (Figure 3A,B). Hydrogen peroxide, known to affect metabolic activity, was used as the positive control. Figure 3 depicts the viability of EA.hy926 cells following a 24 h exposure to CNDs. The results indicate no significant difference in cell viability between the control and CND-treated groups (Figure 3A,B). Flow cytometry plots are read in the following manner: the viable cells are plotted in the bottom left quadrant, and the cells undergoing early apoptosis are in the bottom left quadrant. The top left quadrant is where necrotic cells are plotted, and the top right quadrant is where late apoptotic cells are plotted.

### 3.4. H_2_O_2_ Scavenging Ability of CNDs

**Figure 4:**
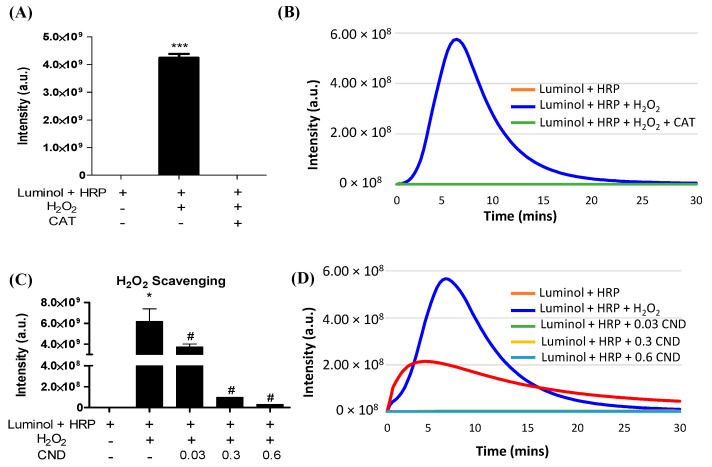
H_2_O_2_ scavenging ability of CNDs: (**A**) the luminol-derived chemiluminescence (CL) is H_2_O_2_ specific. Upon the addition of 500 U/mL catalase, the intensity of CL significantly decreases compared to the experimental group without catalase. (**B**) Real-time changes in CL intensity under catalase action. (**C**) CNDs demonstrate dose-dependent H_2_O_2_ scavenging properties. As the concentration of CNDs increases from 0.03 mg/mL to 0.6 mg/mL, there is a dose-dependent significant decrease in CL intensity. (**D**) Real-time changes in CL intensity under the action of CNDs. *** *p* < 0.001 compared to the Luminol + HRP group. * *p* < 0.05 compared to the Luminol + HRP group (n = 3) and # *p* < 0.05 compared to the Luminol + HRP group + H_2_O_2_ (n = 3).

A luminol-derived chemiluminescence assay was used to study the H_2_O_2_ scavenging ability of CNDs. The chemiluminescence reaction was completely inhibited by catalase, confirming that the assay is H_2_O_2_ specific (Figure 4A,B). The addition of CNDs to the reaction showed a dose-dependent decrease in chemiluminescence compared to the control, indicating that 0.01–0.3 mg/mL CNDs have a strong H_2_O_2_ scavenging capacity (Figure 4C,D).

### 3.5. Effects of CNDs on TNF-α-Induced Expression of Pro-Inflammatory Genes

**Figure 5:**
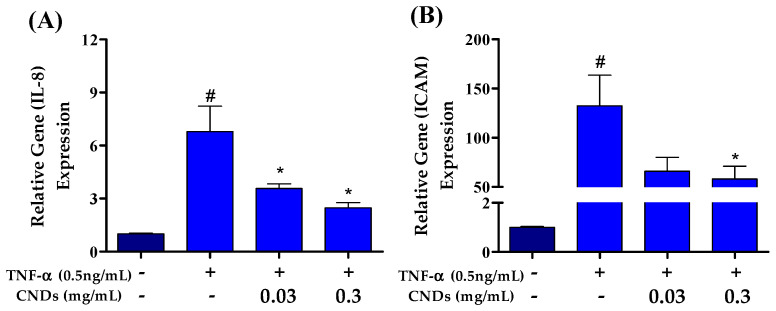
Effects of CNDs on TNF-α-induced expression of pro-inflammatory genes. EA.hy926 cells were co-treated for 24 h with various concentrations of CNDs and 0.5 ng/mL TNF-α. Gene expression was normalized using *GAPDH* as the housekeeping gene. (**A**) Gene expression of *IL-8* genes induced by co-treatments. Our data show a gradual decrease in the target gene expression after co-treatments. (**B**) Gene expression of *ICAM* genes induced by co-treatments. The data shown demonstrate a decrease in expression with increasing CND concentration co-treatments (data are in mean ± SEM (n = 9), # *p* < 0.05 compared to the control group (n = 3) and * *p* < 0.05 compared to the TNF-α group (n = 3).

Atherosclerosis is an inflammatory disease mediated by pro-inflammatory markers. Therefore, we used qRT-PCR to measure CNDs’ effects on TNF-α-induced mRNA levels of several pro-inflammatory genes. EA.hy926 cells were co-treated with 0.5 ng/mL TNF-α and increasing concentrations of CNDs for 24 h. Figure 5A shows a significant decrease in the relative gene expression of the *ICAM* gene following co-treatments with 0.3 mg/mL CNDs compared to TNF-α-induced expression of ICAM. As shown in Figure 5B, there is a significant decrease in mRNA levels of *IL-8* at 0.03 and 0.3 mg/mL concentration of CNDs co-treatments when compared to TNF-α only.

### 3.6. Increase in NOQ1 Activity by CNDs after 24 h

**Figure 6:**
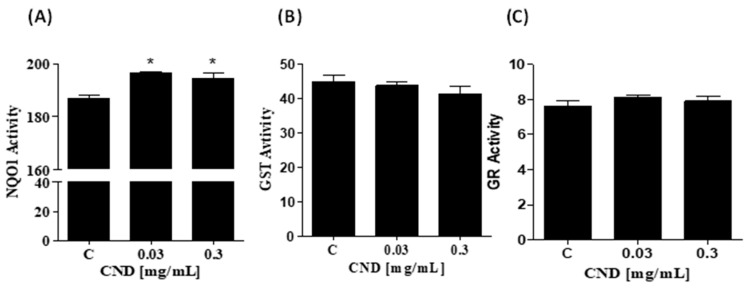
Increase in NOQ1 activity by CNDs after 24 h. Antioxidant lysates were produced after CND exposure for 24 h in HBSS media. (**A**) NQO1 is an enzyme that scavenges for ROS, total activity was measured when compared to the control, 0.03 mg/mL and 0.3 mg/mL CNDs were statistically significant. (**B**) The quantified GST activity for the conditions is not statistically significant compared to the control. (**C**) GR activity in the treatments is similar to the control; GR reduces GSSG to GSH, an important phase II enzyme. Data are in mean ± SEM (n = 3). * *p* < 0.05 compared to the control group (n = 3).

To further examine the intracellular effects of CNDs, we measured the activity of phase II detoxifying enzymes important to xenobiotic biotransformation and excretion. Cells were treated for 24 h with 0 mg/mL, 0 mg/mL, 0.03 mg/mL, and 0.3 mg/mL of CNDs. Samples were prepared to measure the activity of phase II detoxifying enzymes through enzyme kinetic assays. NQO1 activity was significantly increased in 0.03 and 0.3 mg/mL of CND treatments, as seen in Figure 6A. The activity of glutathione s-transferase (Figure 6B) and glutathione reductase (6C) did not differ significantly compared to the control.

## 4. Discussion

In this study, we have demonstrated that the emission peaks of CNDs under different excitations are at about 460 nm, suggesting that the photoluminescence properties of this nanoparticle are excitation independent. Our results are consistent with previous reports that CNDs can be excitation independent according to the synthesis method [27,51,52,53]. This excitation-independent photoluminescence property (Figure 1A,B) allows for analyzing carbon nanodot uptake in EA.hy926 endothelial cells. Using spectrofluorometric measurement, our study demonstrated that CND uptake is both time and concentration dependent in endothelial cells (Figure 2C,D). Carbon nanodot uptake by endothelial cells was further verified through fluorescence microscopy. Currently, there is limited literature evaluating the cellular uptake of CNDs into living cells. In this context, our results present, for the first time, direct evidence that CNDs can enter EA.hy926 endothelial cells at a concentration as low as 0.03 mg/mL. Our research group recently demonstrated that endocytosis involves CND uptake by human microvascular endothelial cells [54]. However, there may be other routes of absorption. Other nanoparticles, such as quantum dots (QDs), may follow different internalization pathways. QDs have been documented to enter HeLa cancer cells through macropinocytosis and cell receptor-mediated endocytosis [55,56]. Due to shared characteristics, whether the entry mechanism of CNDs in the endothelial cells is like that of quantum dots in HeLa cancer cells remains under investigation.

The cell membrane protects intracellular components against the surrounding environment. When xenobiotics enter cells, they may cause toxic or adverse effects if the levels reach high concentrations. Although previous studies have documented the cytotoxicity of CNDs in different cell lines, cytotoxicity has not been analyzed in endothelial cells. Thus, it is important to determine potential toxic concentrations and the mechanism of toxicity of CNDs to endothelial cells. To confirm the effects of CNDs on endothelial cell viability, the Annexin V-FITC with 7-AAD flow cytometry assay was used. Flow cytometry analysis of cell viability showed that the CND concentration used in this experiment did not affect cell viability after 24 h (Figure 3A,B), indicating that the CND dose is safe for endothelial cells.

Previous studies demonstrate that TNF-α plays a fundamental role in endothelial inflammation and initiation of atherosclerosis. This study further analyzed the effect of CNDs on TNFα-induced endothelial inflammation. TNF-α is a homotrimer cell signaling protein, or cytokine, which is known to activate the endothelium, creating an inflammatory response. Pro-inflammatory mediators that play a crucial role in the progression of atherosclerosis are cytokines and surface adhesion molecules such as interleukin 8 (*IL-8*) and intracellular adhesion molecule 1 (*ICAM-1*) [15,16,17,18]. Previous studies show that a decrease in gene expression in *IL-8* can reduce the progression of atherosclerosis [57,58,59]. The expression of *ICAM-1* and other vascular adhesion molecules on the surface of endothelial cells is crucial to the progression of plaque development because of their roles in firm monocyte adhesion to the damaged endothelium. *ICAM-1* can bind to the function-associated antigen (LFA-1) on activated leukocytes; this process helps leukocytes migrate to the tunica intima [60]. Thus, these molecules have been widely accepted as excellent biomarkers of vascular dysfunction [61,62,63,64]. Concurrent with previous studies [65,66], our investigations showed that exposure of EA.hy926 endothelial cells to TNF-α significantly induced the expression of *IL-8* and *ICAM-1*, indicating the critical role of these chemokines/adhesion molecules in TNF-α-induced vascular inflammation. TNF-α-induced *IL-8* expression is decreased significantly with increasing CND exposure, suggesting the decrease in *IL-8* to activate and recruit leukocytes to the site of inflammation. Likewise, CND concentrations as low as 0.3 mg/mL also inhibited TNF-α-induced *ICAM-1* expression. These results demonstrated the anti-inflammatory effect of CNDs in a dose-dependent manner.

Studies have shown that reactive oxygen species (ROS) such as hydrogen peroxide ROS (such as hydrogen) play an important role in TNF-α-induced inflammation [67,68,69,70]. These reactive oxygen species have been shown to regulate TNF-α by activating various pro-inflammatory cytokines [71]. Luminol has been widely used as a chemiluminescent probe for detecting hydrogen peroxide levels under different experimental conditions. In this system, luminol-derived chemiluminescence in the presence of horseradish peroxidase is known to detect H_2_O_2_ production. The results of our chemiluminometric assay indicate the H_2_O_2_ scavenging ability of CNDs (0.03–0.3 mg/mL) increased dose dependently (Figure 4). These data suggest that CNDs have direct scavenging activity against H_2_O_2_, which may partially explain their anti-inflammatory effects in vitro. It is unclear whether TNF-α can directly stimulate EA.hy926 endothelial cells to stimulate hydrogen peroxide production in mitochondria. Previous research demonstrated that complexes I and III of the mitochondrial electron transport chain can generate superoxide radicals. Mitochondrial complex I has been shown to release superoxide specifically into the mitochondrial matrix, while complex III releases superoxide to the matrix and intermembrane space [72]. Superoxide radicals are then detoxified by mitochondrial superoxide dismutase (SOD2), producing hydrogen peroxide [73,74,75,76].

We further investigated whether the anti-inflammatory effects of CNDs are mediated through other redox mechanisms, such as the induction of endogenous antioxidants and phase 2 enzymes in endothelial cells. Important antioxidants in the vasculature that protect against oxidative stress by quenching ROS include NQO1, GST, and GR [77]. NQO1 is a cytoprotective enzyme; one of its many known functions consists of the 2 electron reduction of quinines, along with decreasing the radical formation of semiquinones and the resulting ROS [78]. An additional function is to sustain the levels of important antioxidants like ubiquinone and vitamin E by reducing their derivatives to antioxidants [79,80]. It has also been established that NQO1 scavenges superoxide anion radicals, protecting vasculature from oxidative stress [78,81]. Interestingly, studies have shown that NQO1 is expressed at much higher levels in cardiovascular tissue than in other tissue types [82]. Therefore, we explored whether CND treatment could increase the activities of NQO1 in cultured EA.hy926 endothelial cells. Interestingly, our results show that only the activity of NQO1 increased (Figure 6A). In agreement with previous studies outlining the significant role of NQO1 in the maintenance of vascular homeostasis [83], our data suggest that NQO1 may be a cytoprotective mechanism of CNDs to protect against TNF-α-induced endothelial inflammation. We have previously reported that NQO1 levels are much higher in cardiovascular cells, including endothelial cells, smooth muscle, and cardiac H9c2 cells, than in other cell types, including bone marrow stromal cells, human SH-SY5Y neuronal cells, and human Caco-2 carcinomas [82,84]. It has been demonstrated that purified NAD(P)H:quinone oxidoreductase 1 (NQO1) can scavenge superoxide, and that this occurs only when NQO1 levels are high and superoxide dismutase (SOD) levels are low [82,84,85]. Future studies should examine SOD levels in Ea.Hy926 cells to explore the relationship between changes in SOD levels and upregulation of NQO1 enzyme content by CNDs (Figure 6). In our previous study on HMEC-1 cells [54], cells were treated with CNDs for 6 h, and a significant increase in *HO-1* gene expression, but not NQO1, was detected using qPCR. In the current study, EA.hy926 cells treated with CNDs for 24 h significantly increased NQO1 activity in EA.hy926 endothelial cells (Figure 6), which may be due to the longer exposure time. The possible reason why CNDs did not increase *NQO1* gene expression in our previous study [54] in HMEC cells after 6 h of exposure was that the exposure time was short. It is also important to note that EA.hy926 and HMEC-1 cell lines have distinct transcriptomic profiles [86] and previous studies have suggested that these endothelial cells may have similar phenotypes but may respond differently to compounds such as the senolytic drug ABT-263 [87]. However, it is unclear how CNDs increase cellular NQO1 activity in endothelial cells. Activation of nuclear factor erythroid 2-related factor 2 (Nrf2)/Keap-1/ARE signaling has been proposed in concurrent studies to play an essential role in the expression of intracellular NQO-1 [88]. Further investigation is required to examine the nuclear translocation of Nrf2 to understand the effect of CNDs on NQO1 enzyme activity. Understanding the mechanism of action by which CNDs activate NOQ1 is essential due to the role of NOQ1 in regulating healthy vascular homeostasis.

In our previous study on HMEC-1 cells [54], CNDs at concentrations ranging from 0.1–0.3 mg/mL were found to reduce TNF-α-induced endothelial inflammation. However, high concentrations of TNF-α (10 ng/mL) were used to induce changes in pro-inflammatory biomarkers in HMEC-1. In this study, a very low concentration of TNF-α (0.5 ng/mL) was used. Many studies have reported that levels of TNFα in human plasma from patients with various inflammation-induced diseases [89,90,91] are well below the level (10 ng/mL) used to elicit maximal responses in many experimental studies [92,93,94,95,96,97,98,99]. Therefore, the results of this study of CNDs on TNF-α at a low concentration of 0.5 ng/mL may have more clinical significance. Furthermore, the exposure times of CNDs in the two different cell models were completely different. In previous studies on HMEC-1 cells, CND treatment was performed for a short period of only 6 h. This study exposed Eahy.926 cells to CNDs for a longer period of time, 24 h. In addition, this study is the first to report that CNDs have H_2_O_2_ scavenging ability, and its scavenging ability increases in a dose-dependent manner, indicating that CNDs have direct scavenging activity on H_2_O_2_, which may partially explain its in vitro anti-inflammatory effect.

## 5. Conclusions

In summary, our study provided a biological characterization of CNDs in EA.hy926 endothelial cells. Utilizing the intrinsic fluorescence of CNDs, it was found that incubation of EA.hy926 cells with CNDs resulted in a dose and time-dependent intracellular uptake of this nanoparticle. The concentration of CNDs used in this experiment was found to not affect cell viability. CNDs as low as 0.03 mg/mL significantly inhibited TNF-α-mediated expression of *IL-8* and adhesion molecule *ICAM-1*, two key molecules that are responsible for the activation and the firm adhesion of monocytes to activated endothelial cells for the initiation of atherosclerosis. ROS, such as hydrogen peroxide, plays an important role in TNF-α-induced inflammation. Interestingly, we found that CNDs as low as 0.03 mg/mL effectively scavenged H_2_O_2_ in a dose-dependent manner, suggesting that the anti-inflammatory effects of CNDs are likely due to their ability to scavenge H_2_O_2_ directly. CND treatment also increased the activity of the antioxidant enzyme NQO1 in EA.hy926 endothelial cells. These results suggest that the anti-inflammatory effects of CNDs may be due to the direct H_2_O_2_ scavenging properties of CNDs and the indirect upregulation of NQO1 activity in endothelial cells. However, the exact molecular mechanisms involved are largely unknown and remain to be investigated. The possible impact of CNDs on vascular inflammation may provide new information on the future application of CNDs as an effective treatment for inflammatory disorders such as atherosclerosis.

## Data Availability

The data used to support the findings of this study are available from the corresponding author upon reasonable request.

## References

[1] WHF Cardiovascular Diseases Infographic. https://world-heart-federation.org/resource/cardiovascular-disease-infographic/.

[2] WebMD Cardiovascular Diseases. https://www.webmd.com/heart-disease/guide/diseases-cardiovascular.

[3] Damluji A.A., Alfaraidhy M., AlHajri N., Rohant N.N., Kumar M., Al Malouf C., Bahrainy S., Ji Kwak M., Batchelor W.B., Forman D.E. (2023). Sarcopenia and Cardiovascular Diseases. Circulation.

[4] Dimopoulos K., Constantine A., Clift P., Condliffe R., Moledina S., Jansen K., Inuzuka R., Veldtman G.R., Cua C.L., Tay E.L.W. (2023). Cardiovascular Complications of Down Syndrome: Scoping Review and Expert Consensus. Circulation.

[5] Musunuru K. (2023). CRISPR and cardiovascular diseases. Cardiovasc. Res..

[6] Valenzuela P.L., Ruilope L.M., Santos-Lozano A., Wilhelm M., Kränkel N., Fiuza-Luces C., Lucia A. (2023). Exercise benefits in cardiovascular diseases: From mechanisms to clinical implementation. Eur. Heart J..

[7] CDC Heart Disease Facts. https://www.cdc.gov/heartdisease/facts.htm.

[8] Go A.S., Mozaffarian D., Roger V.L., Benjamin E.J., Berry J.D., Blaha M.J., Dai S., Ford E.S., Fox C.S., Franco S. (2014). Heart disease and stroke statistics--2014 update: A report from the American Heart Association. Circulation.

[9] Benjamin E.J., Blaha M.J., Chiuve S.E., Cushman M., Das S.R., Deo R., de Ferranti S.D., Floyd J., Fornage M., Gillespie C. (2017). Heart Disease and Stroke Statistics-2017 Update: A Report From the American Heart Association. Circulation.

[10] Medicine J.H. Atherosclerosis. https://www.hopkinsmedicine.org/health/conditions-and-diseases/atherosclerosis.

[11] Ait-Oufella H., Taleb S., Mallat Z., Tedgui A. (2011). Recent advances on the role of cytokines in atherosclerosis. Arter. Thromb. Vasc. Biol..

[12] Doran A.C. (2022). Inflammation Resolution: Implications for Atherosclerosis. Circ. Res..

[13] Libby P. (2021). The changing landscape of atherosclerosis. Nature.

[14] Pedro-Botet J., Climent E., Benaiges D. (2020). Atherosclerosis and inflammation. New therapeutic approaches. Med. Clin..

[15] Pearson T.A., Mensah G.A., Alexander R.W., Anderson J.L., Cannon R.O., Criqui M., Fadl Y.Y., Fortmann S.P., Hong Y., Myers G.L. (2003). Markers of inflammation and cardiovascular disease: Application to clinical and public health practice: A statement for healthcare professionals from the Centers for Disease Control and Prevention and the American Heart Association. Circulation.

[16] Thompson S.G., Kienast J., Pyke S.D., Haverkate F., van de Loo J.C. (1995). Hemostatic factors and the risk of myocardial infarction or sudden death in patients with angina pectoris. European Concerted Action on Thrombosis and Disabilities Angina Pectoris Study Group. N. Engl. J. Med..

[17] Danesh J., Wheeler J.G., Hirschfield G.M., Eda S., Eiriksdottir G., Rumley A., Lowe G.D., Pepys M.B., Gudnason V. (2004). C-reactive protein and other circulating markers of inflammation in the prediction of coronary heart disease. N. Engl. J. Med..

[18] Ridker P.M., Hennekens C.H., Buring J.E., Rifai N. (2000). C-reactive protein and other markers of inflammation in the prediction of cardiovascular disease in women. N. Engl. J. Med..

[19] Khan S., Dunphy A., Anike M.S., Belperain S., Patel K., Chiu N.H., Jia Z. (2021). Recent advances in carbon nanodots: A promising nanomaterial for biomedical applications. Int. J. Mol. Sci..

[20] Hoshino A., Manabe N., Fujioka K., Suzuki K., Yasuhara M., Yamamoto K. (2007). Use of fluorescent quantum dot bioconjugates for cellular imaging of immune cells, cell organelle labeling, and nanomedicine: Surface modification regulates biological function, including cytotoxicity. J. Artif. Organs.

[21] Lin L., Song X., Chen Y., Rong M., Zhao T., Wang Y., Jiang Y., Chen X. (2015). Intrinsic peroxidase-like catalytic activity of nitrogen-doped graphene quantum dots and their application in the colorimetric detection of H_2_O_2_ and glucose. Anal. Chim. Acta.

[22] Miao P., Han K., Tang Y., Wang B., Lin T., Cheng W. (2015). Recent advances in carbon nanodots: Synthesis, properties and biomedical applications. Nanoscale.

[23] Chan M.H., Chen B.G., Ngo L.T., Huang W.T., Li C.H., Liu R.S., Hsiao M. (2021). Natural Carbon Nanodots: Toxicity Assessment and Theranostic Biological Application. Pharmaceutics.

[24] Cui L., Ren X., Sun M., Liu H., Xia L. (2021). Carbon Dots: Synthesis, Properties and Applications. Nanomaterials.

[25] Kong B., Zhu A., Ding C., Zhao X., Li B., Tian Y. (2012). Carbon dot-based inorganic-organic nanosystem for two-photon imaging and biosensing of pH variation in living cells and tissues. Adv. Mater..

[26] Sharma A., Gadly T., Gupta A., Ballal A., Ghosh S.K., Kumbhakar M. (2016). Origin of Excitation Dependent Fluorescence in Carbon Nanodots. J. Phys. Chem. Lett..

[27] Zhu S., Meng Q., Wang L., Zhang J., Song Y., Jin H., Zhang K., Sun H., Wang H., Yang B. (2013). Highly photoluminescent carbon dots for multicolor patterning, sensors, and bioimaging. Angew. Chem. Int. Ed..

[28] Chen P.C., Chen Y.N., Hsu P.C., Shih C.C., Chang H.T. (2013). Photoluminescent organosilane-functionalized carbon dots as temperature probes. Chem. Commun..

[29] Yang Y., Cui J., Zheng M., Hu C., Tan S., Xiao Y., Yang Q., Liu Y. (2012). One-step synthesis of amino-functionalized fluorescent carbon nanoparticles by hydrothermal carbonization of chitosan. Chem. Commun..

[30] Kong D., Yan F., Luo Y., Wang Y., Chen L., Cui F. (2016). Carbon nanodots prepared for cellular imaging and turn-on detection of glutathione. Anal. Methods.

[31] Tang J., Kong B., Wu H., Xu M., Wang Y., Wang Y., Zhao D., Zheng G. (2013). Carbon nanodots featuring efficient FRET for real-time monitoring of drug delivery and two-photon imaging. Adv. Mater..

[32] Wang S., Li C., Qian M., Jiang H., Shi W., Chen J., Lachelt U., Wagner E., Lu W., Wang Y. (2017). Augmented glioma-targeted theranostics using multifunctional polymer-coated carbon nanodots. Biomaterials.

[33] Xu Z.Q., Lan J.Y., Jin J.C., Dong P., Jiang F.L., Liu Y. (2015). Highly Photoluminescent Nitrogen-Doped Carbon Nanodots and Their Protective Effects against Oxidative Stress on Cells. ACS Appl. Mater. Interfaces.

[34] Bankoti K., Rameshbabu A.P., Datta S., Das B., Mitra A., Dhara S. (2017). Onion derived carbon nanodots for live cell imaging and accelerated skin wound healing. J. Mater. Chem. B.

[35] Zhang W., Zeng Z., Wei J. (2017). Electrochemical Study of DPPH Radical Scavenging for Evaluating the Antioxidant Capacity of Carbon Nanodots. J. Phys. Chem. C.

[36] Kato K., Terao S., Shimamoto N., Hirata M. (1988). Studies on scavengers of active oxygen species. 1. Synthesis and biological activity of 2-O-alkylascorbic acids. J. Med. Chem..

[37] Parveen M., Ahmad F., Malla A.M., Azaz S. (2016). Microwave-assisted green synthesis of silver nanoparticles from Fraxinus excelsior leaf extract and its antioxidant assay. Appl. Nanosci..

[38] Blois M.S. (1958). Antioxidant Determinations by the Use of a Stable Free Radical. Nature.

[39] Levine B., Kalman J., Mayer L., Fillit H.M., Packer M. (1990). Elevated circulating levels of tumor necrosis factor in severe chronic heart failure. N. Engl. J. Med..

[40] Hallenbeck J.M. (2002). The many faces of tumor necrosis factor in stroke. Nat. Med..

[41] Wang X., Zhang R., Gu L., Zhang Y., Zhao X., Bi K., Chen X. (2015). Cell-based screening identifies the active ingredients from Traditional Chinese Medicine formula Shixiao San as the inhibitors of atherosclerotic endothelial dysfunction. PLoS ONE.

[42] Zhu M., Li J., Wang K., Hao X., Ge R., Li Q. (2016). Isoquercitrin Inhibits Hydrogen Peroxide-Induced Apoptosis of EA.hy926 Cells via the PI3K/Akt/GSK3β Signaling Pathway. Molecules.

[43] Shi J., Chen D., Wang Z., Li S., Zhang S. (2023). Homocysteine induces ferroptosis in endothelial cells through the systemXc(-)/GPX4 signaling pathway. BMC Cardiovasc. Disord..

[44] Dunphy A., Patel K., Belperain S., Pennington A., Chiu N.H.L., Yin Z., Zhu X., Priebe B., Tian S., Wei J. (2021). Modulation of Macrophage Polarization by Carbon Nanodots and Elucidation of Carbon Nanodot Uptake Routes in Macrophages. Nanomaterials.

[45] Ji Z., Yin Z., Jia Z., Wei J. (2020). Carbon Nanodots Derived from Urea and Citric Acid in Living Cells: Cellular Uptake and Antioxidation Effect. Langmuir.

[46] Shukla H., Chitrakar R., Bibi H.A., Gaje G., Koucheki A., Trush M.A., Zhu H., Li Y.R., Jia Z. (2020). Reactive oxygen species production by BP-1,6-quinone and its effects on the endothelial dysfunction: Involvement of the mitochondria. Toxicol. Lett..

[47] Siddique A.B., Pramanick A.K., Chatterjee S., Ray M. (2018). Amorphous Carbon Dots and their Remarkable Ability to Detect 2,4,6-Trinitrophenol. Sci. Rep..

[48] Pal A., Sk M.P., Chattopadhyay A. (2020). Recent advances in crystalline carbon dots for superior application potential. Mater. Adv..

[49] Zhang W., Chavez J., Zeng Z., Bloom B., Sheardy A., Ji Z., Yin Z., Waldeck D.H., Jia Z., Wei J. (2018). Antioxidant capacity of nitrogen and sulfur codoped carbon nanodots. ACS Appl. Nano Mater..

[50] Wikipedia Hydrooxide. https://en.wikipedia.org/wiki/Hydroxide.

[51] Liu H., He Z., Jiang L.P., Zhu J.J. (2015). Microwave-assisted synthesis of wavelength-tunable photoluminescent carbon nanodots and their potential applications. ACS Appl. Mater. Interfaces.

[52] Liu J., Lu S., Tang Q., Zhang K., Yu W., Sun H., Yang B. (2017). One-step hydrothermal synthesis of photoluminescent carbon nanodots with selective antibacterial activity against *Porphyromonas gingivalis*. Nanoscale.

[53] Park S.Y., Lee H.U., Park E.S., Lee S.C., Lee J.W., Jeong S.W., Kim C.H., Lee Y.C., Huh Y.S., Lee J. (2014). Photoluminescent green carbon nanodots from food-waste-derived sources: Large-scale synthesis, properties, and biomedical applications. ACS Appl. Mater. Interfaces.

[54] Belperain S., Kang Z.Y., Dunphy A., Priebe B., Chiu N.H., Jia Z. (2021). Anti-inflammatory effect and cellular uptake mechanism of carbon nanodots in in human microvascular endothelial cells. Nanomaterials.

[55] Ruan G., Agrawal A., Marcus A.I., Nie S. (2007). Imaging and tracking of tat peptide-conjugated quantum dots in living cells: New insights into nanoparticle uptake, intracellular transport, and vesicle shedding. J. Am. Chem. Soc..

[56] Osaki F., Kanamori T., Sando S., Sera T., Aoyama Y. (2004). A quantum dot conjugated sugar ball and its cellular uptake. On the size effects of endocytosis in the subviral region. J. Am. Chem. Soc..

[57] Apostolakis S., Vogiatzi K., Amanatidou V., Spandidos D.A. (2009). Interleukin 8 and cardiovascular disease. Cardiovasc. Res..

[58] Latruffe N., Lancon A., Frazzi R., Aires V., Delmas D., Michaille J.J., Djouadi F., Bastin J., Cherkaoui-Malki M. (2015). Exploring new ways of regulation by resveratrol involving miRNAs, with emphasis on inflammation. Ann. N. Y. Acad. Sci..

[59] Rezaie-Majd A., Maca T., Bucek R.A., Valent P., Muller M.R., Husslein P., Kashanipour A., Minar E., Baghestanian M. (2002). Simvastatin reduces expression of cytokines interleukin-6, interleukin-8, and monocyte chemoattractant protein-1 in circulating monocytes from hypercholesterolemic patients. Arter. Thromb. Vasc. Biol..

[60] Kurokouchi K., Kambe F., Yasukawa K., Izumi R., Ishiguro N., Iwata H., Seo H. (1998). TNF-alpha increases expression of IL-6 and ICAM-1 genes through activation of NF-kappaB in osteoblast-like ROS17/2.8 cells. J. Bone Min. Res..

[61] Hansson G.K. (1994). Immune and inflammatory mechanisms in the pathogenesis of atherosclerosis. J. Atheroscler. Thromb..

[62] Libby P., Ridker P.M., Maseri A. (2002). Inflammation and atherosclerosis. Circulation.

[63] Mangge H., Becker K., Fuchs D., Gostner J.M. (2014). Antioxidants, inflammation and cardiovascular disease. World J. Cardiol..

[64] Collins T. (1993). Endothelial nuclear factor-kappa B and the initiation of the atherosclerotic lesion. Lab. Investig..

[65] Tak P.P., Firestein G.S. (2001). NF-kappaB: A key role in inflammatory diseases. J. Clin. Investig..

[66] Pamukcu B., Lip G.Y., Devitt A., Griffiths H., Shantsila E. (2010). The role of monocytes in atherosclerotic coronary artery disease. Ann. Med..

[67] Hansson G.K. (2005). Inflammation, atherosclerosis, and coronary artery disease. N. Engl. J. Med..

[68] Ross R. (1999). Atherosclerosis--an inflammatory disease. N. Engl. J. Med..

[69] Steinberg D. (2002). Atherogenesis in perspective: Hypercholesterolemia and inflammation as partners in crime. Nat. Med..

[70] Radeke H.H., Meier B., Topley N., Floge J., Habermehl G.G., Resch K. (1990). Interleukin 1-alpha and tumor necrosis factor-alpha induce oxygen radical production in mesangial cells. Kidney Int..

[71] Gloire G., Legrand-Poels S., Piette J. (2006). NF-kappaB activation by reactive oxygen species: Fifteen years later. Biochem. Pharmacol..

[72] Dröse S., Brandt U. (2012). Molecular mechanisms of superoxide production by the mitochondrial respiratory chain. Adv. Exp. Med. Biol..

[73] Guo Y., Guan T., Shafiq K., Yu Q., Jiao X., Na D., Li M., Zhang G., Kong J. (2023). Mitochondrial dysfunction in aging. Ageing Res. Rev..

[74] Kidwell C.U., Casalini J.R., Pradeep S., Scherer S.D., Greiner D., Bayik D., Watson D.C., Olson G.S., Lathia J.D., Johnson J.S. (2023). Transferred mitochondria accumulate reactive oxygen species, promoting proliferation. eLife.

[75] Su L., Zhang J., Gomez H., Kellum J.A., Peng Z. (2023). Mitochondria ROS and mitophagy in acute kidney injury. Autophagy.

[76] Turrens J.F. (2003). Mitochondrial formation of reactive oxygen species. J. Physiol..

[77] Stocker R., Keaney J.F. (2004). Role of oxidative modifications in atherosclerosis. Physiol. Rev..

[78] Ross D., Kepa J.K., Winski S.L., Beall H.D., Anwar A., Siegel D. (2000). NAD(P)H:quinone oxidoreductase 1 (NQO1): Chemoprotection, bioactivation, gene regulation and genetic polymorphisms. Chem. Biol. Interact..

[79] Ross D. (2004). Quinone reductases multitasking in the metabolic world. Drug Metab. Rev..

[80] Siegel D., Bolton E.M., Burr J.A., Liebler D.C., Ross D. (1997). The reduction of alpha-tocopherolquinone by human NAD(P)H: Quinone oxidoreductase: The role of alpha-tocopherolhydroquinone as a cellular antioxidant. Mol. Pharmacol..

[81] Zhao Q., Yang X.L., Holtzclaw W.D., Talalay P. (1997). Unexpected genetic and structural relationships of a long-forgotten flavoenzyme to NAD(P)H:quinone reductase (DT-diaphorase). Proc. Natl. Acad. Sci. USA.

[82] Zhu H., Jia Z., Mahaney J.E., Ross D., Misra H.P., Trush M.A., Li Y. (2007). The highly expressed and inducible endogenous NAD(P)H:quinone oxidoreductase 1 in cardiovascular cells acts as a potential superoxide scavenger. Cardiovasc. Toxicol..

[83] Isbir C.S., Ergen A., Tekeli A., Zeybek U., Gormus U., Arsan S. (2008). The effect of NQO1 polymorphism on the inflammatory response in cardiopulmonary bypass. Cell Biochem. Funct..

[84] Shukla H., Gaje G., Koucheki A., Lee H.Y., Sun X., Trush M.A., Zhu H., Li Y.R., Jia Z. (2020). NADPH-quinone oxidoreductase-1 mediates Benzo-[a]-pyrene-1,6-quinone-induced cytotoxicity and reactive oxygen species production in human EA.hy926 endothelial cells. Toxicol. Appl. Pharmacol..

[85] Ross D., Siegel D. (2018). NQO1 in protection against oxidative stress. Curr. Opin. Toxicol..

[86] Ma X., Sickmann A., Pietsch J., Wildgruber R., Weber G., Infanger M., Bauer J., Grimm D. (2014). Proteomic differences between microvascular endothelial cells and the EA.hy926 cell line forming three-dimensional structures. Proteomics.

[87] Abdelgawad I.Y., Agostinucci K., Ismail S.G., Grant M.K.O., Zordoky B.N. (2022). EA.hy926 Cells and HUVECs Share Similar Senescence Phenotypes but Respond Differently to the Senolytic Drug ABT-263. Cells.

[88] Itoh K., Wakabayashi N., Katoh Y., Ishii T., O’Connor T., Yamamoto M. (2003). Keap1 regulates both cytoplasmic-nuclear shuttling and degradation of Nrf2 in response to electrophiles. Genes Cells.

[89] Bolajoko E.B., Arinola O.G., Odaibo G.N., Maiga M. (2020). Plasma levels of tumor necrosis factor-alpha, interferon-gamma, inducible nitric oxide synthase, and 3-nitrotyrosine in drug-resistant and drug-sensitive pulmonary tuberculosis patients, Ibadan, Nigeria. Int. J. Mycobacteriol..

[90] Nakai Y., Hamagaki S., Takagi R., Taniguchi A., Kurimoto F. (1999). Plasma concentrations of tumor necrosis factor-alpha (TNF-alpha) and soluble TNF receptors in patients with anorexia nervosa. J. Clin. Endocrinol. Metab..

[91] Turner D.A., Paszek P., Woodcock D.J., Nelson D.E., Horton C.A., Wang Y., Spiller D.G., Rand D.A., White M.R., Harper C.V. (2010). Physiological levels of TNFalpha stimulation induce stochastic dynamics of NF-kappaB responses in single living cells. J. Cell Sci..

[92] Damas P., Reuter A., Gysen P., Demonty J., Lamy M., Franchimont P. (1989). Tumor necrosis factor and interleukin-1 serum levels during severe sepsis in humans. Crit. Care Med..

[93] Nallasamy P., Kang Z.Y., Sun X., Anandh Babu P.V., Liu D., Jia Z. (2021). Natural Compound Resveratrol Attenuates TNF-Alpha-Induced Vascular Dysfunction in Mice and Human Endothelial Cells: The Involvement of the NF-κB Signaling Pathway. Int. J. Mol. Sci..

[94] Boutagy N.E., Fowler J.W., Grabinska K.A., Cardone R., Sun Q., Vazquez K.R., Whalen M.B., Zhu X., Chakraborty R., Martin K.A. (2023). TNFα increases the degradation of pyruvate dehydrogenase kinase 4 by the Lon protease to support proinflammatory genes. Proc. Natl. Acad. Sci. USA.

[95] Festa J., Hussain A., Hackney A., Desai U., Sahota T.S., Singh H., Da Boit M. (2023). Elderberry extract improves molecular markers of endothelial dysfunction linked to atherosclerosis. Food Sci. Nutr..

[96] Guan S., Sun L., Wang X., Huang X., Luo T. (2023). Propofol inhibits neuroinflammation and metabolic reprogramming in microglia in vitro and in vivo. Front. Pharmacol..

[97] Janpaijit S., Sillapachaiyaporn C., Theerasri A., Charoenkiatkul S., Sukprasansap M., Tencomnao T. (2023). Cleistocalyx nervosum var. paniala Berry Seed Protects against TNF-α-Stimulated Neuroinflammation by Inducing HO-1 and Suppressing NF-κB Mechanism in BV-2 Microglial Cells. Molecules.

[98] Leisegang K., Finelli R., Henkel R., Zenoaga-Barbăroșie C. (2023). The Effect of Aqueous Lessertia frutescens Extract on TM3 Leydig Cells Exposed to TNF-α in vitro. Front. Biosci. Landmark Ed..

[99] Palafox-Mariscal L.A., Ortiz-Lazareno P.C., Jave-Suárez L.F., Aguilar-Lemarroy A., Villaseñor-García M.M., Cruz-Lozano J.R., González-Martínez K.L., Méndez-Clemente A.S., Bravo-Cuellar A., Hernández-Flores G. (2023). Pentoxifylline Inhibits TNF-α/TGF-β1-Induced Epithelial-Mesenchymal Transition via Suppressing the NF-κB Pathway and SERPINE1 Expression in CaSki Cells. Int. J. Mol. Sci..

